# Supplementary material for: Evaluation of MALDI-ToF Mass Spectrometry for Rapid Detection of Cereulide From Bacillus cereus Cultures
Source: Front Microbiol. 2020 Oct 6;11:511674. doi: 10.3389/fmicb.2020.511674 (PMC7709880; doi:10.3389/fmicb.2020.511674)
Supplement: Supplementary file 1 [file Data_Sheet_1.PDF]

Evaluation of MALDI-ToF Mass Spectrometry for Rapid Detection of Cereulide from *Bacillus cereus* Cultures

Joerg Doellinger[1], Andy Schneider[1] Timo D. Stark[2], Monika Ehling-Schulz[3] and Peter Lasch[1]

[1] Proteomics and Spectroscopy unit (ZBS 6) at the Centre for Biological Threats and Special Pathogens, Robert Koch-Institute, Seestraße 10, D-13353 Berlin, Germany
[2] Technical University of Munich, Food Chemistry and Molecular Sensory Science, 85354 Freising, Lise-Meitner-Straße 34, Munich, Germany
[3] Functional Microbiology, Institute of Microbiology, Department of Pathobiology, University of Veterinary Medicine Vienna, 1210, Vienna, Austria

Supporting Information

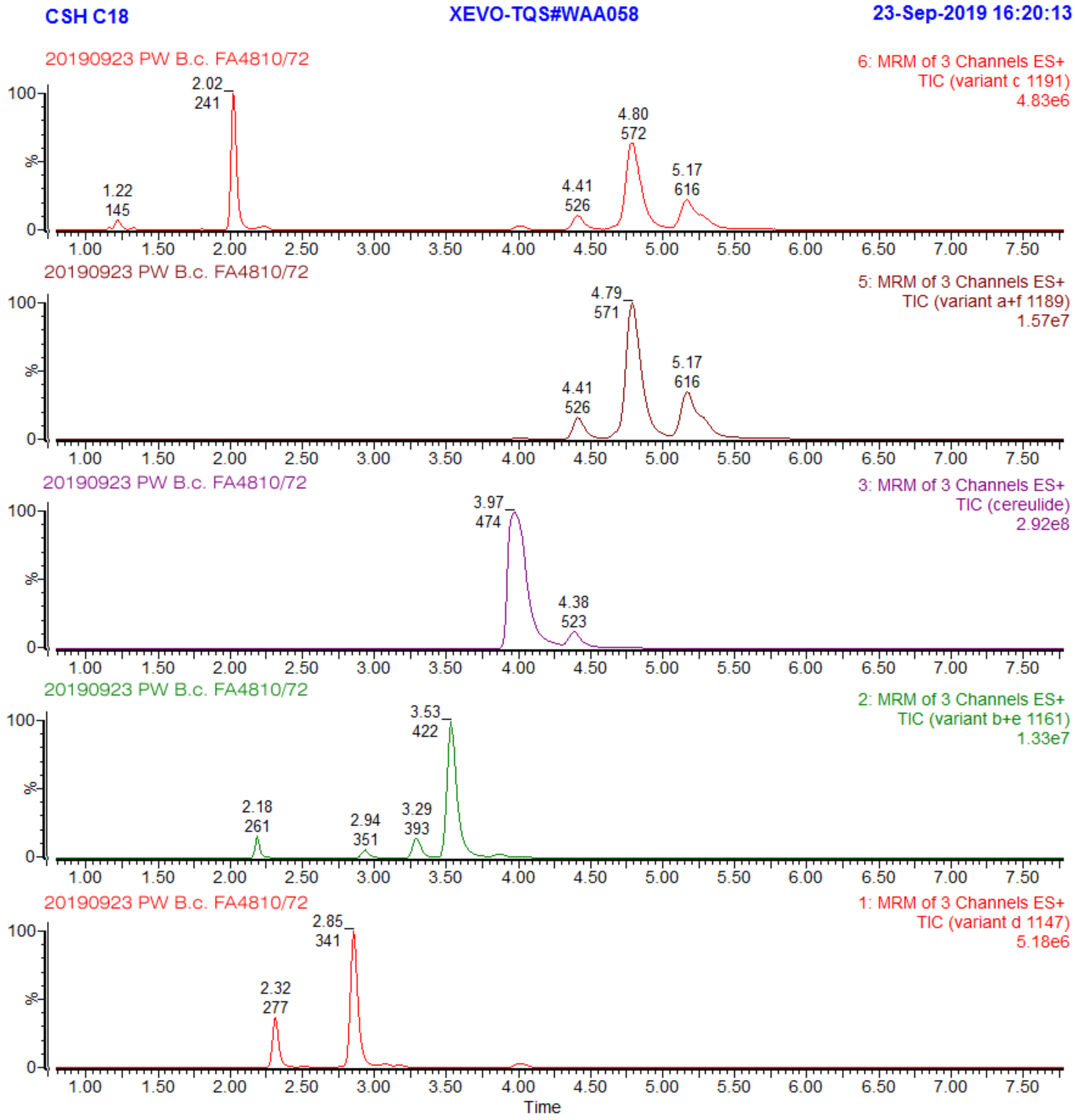
CSH C18
XEVO-TQS#WAA058
23-Sep-2019 16:20:13
20190923 PW B.c. FA4810/72
6: MRM of 3 Channels ES+
TIC (variant c 1191)
4.83e6
5: MRM of 3 Channels ES+
TIC (variant a+f 1189)
1.57e7
3: MRM of 3 Channels ES+
TIC (cereulide)
2.92e8
2: MRM of 3 Channels ES+
TIC (variant b+e 1161)
1.33e7
1: MRM of 3 Channels ES+
TIC (variant d 1147)
5.18e6
Time

Figure SI-01. Ultraperformance Liquid Chromatography – Mass Spectrometry (UPLC-MS/MS) Multiple Reaction Monitoring (MRM) Analysis of ethanolic washing solutions of *B.cereus* F4810/72.

Mass spectrometric analyses were performed on a Waters Xevo TQ-S mass spectrometer (Waters, Manchester, UK) coupled to an Acquity UPLC i-class core system (Waters, Milford, MA, USA) consisting of a binary solvent manager, sample manager, and column oven. Aliquots (2 μL) of prepared samples were injected into the UPLC/MS-MS system equipped with a 2.1 x 150 mm, 1.7 μm, UPLC CSH C18 column (Waters, Manchester, UK). Operated with a flow rate of 0.7 mL/min at a temperature of 55 °C, the following gradient was used for chromatography: starting with a mixture (85/15, v/v) of MeCN (0.25 % HCOOH) as solvent A and $HCOONH_4$ (10 mmol, 0.1 % HCOOH) as solvent B, solvent A was increased to 95 % within 8.0 min, increased to 99 % within 0.1 min, kept constant for 0.9 min, decreased within 0.1 min to 85 %, and followed by re-equilibration on starting conditions for 0.9 min. Measurements were performed using positive electrospray ionization (ESI) and the quantitative calibration mode consisting of the following ion source parameters: capillary voltage +3.6 kV, sampling cone 50 V, source offset 35 V, source temperature 150 °C, desolvation temperature 650 °C, cone gas 250 L/h, desolvation gas 1100 L/h, collision gas flow 0.15 mL/min and nebuliser gas flow 7.0 bar. Calibration of the mass spectrometer in the range from *m/z* 40-1963 was performed using a solution of phosphoric acid (0.1 % in MeCN). The UPLC Xevo TQ-S system was operated with MassLynx^{TM} 4.1 SCN 813 (Waters), data processing and analysis were performed using TargetLynx (Waters). By means of the multiple reaction monitoring (MRM) mode, the ammonium adducts of cereulide and isocereulides were analyzed using the mass transitions given inthe Table below monitored for a duration of 25 ms. ESI$^+$ mass and product ion spectra were acquired with direct flow infusion using IntelliStart. The MS/MS parameters were tuned for each individual compound, detecting the fragmentation of the $[M+NH_4]^+$ molecular ions into specific product ions after collision with argon.

Table summarizing the MRM transitions

isocereulide G: *m/z* 1170.7 → qualifier: *m/z* 172.2, 314.2; quantifier: *m/z* 357.2

isocereulide A and F: *m/z* 1184.7 → qualifier: *m/z* 172.2, 314.2; quantifier: *m/z* 357.2

isocereulide B and E: *m/z* 1156.6 → qualifier: *m/z* 172.2, 314.2; quantifier: *m/z* 357.2

isocereulide C: *m/z* 1186.6 → qualifier: *m/z* 172.2, 314.2; quantifier: *m/z* 357.2

isocereulide D: *m/z* 1142.6 → qualifier: *m/z* 172.2, 314.2; quantifier: *m/z* 357.2

$^{13}C_6$-cereulide (*m/z* 1176.7 → m/z qualifier: 173.2, 316.2; quantifier: *m/z* 358.2)

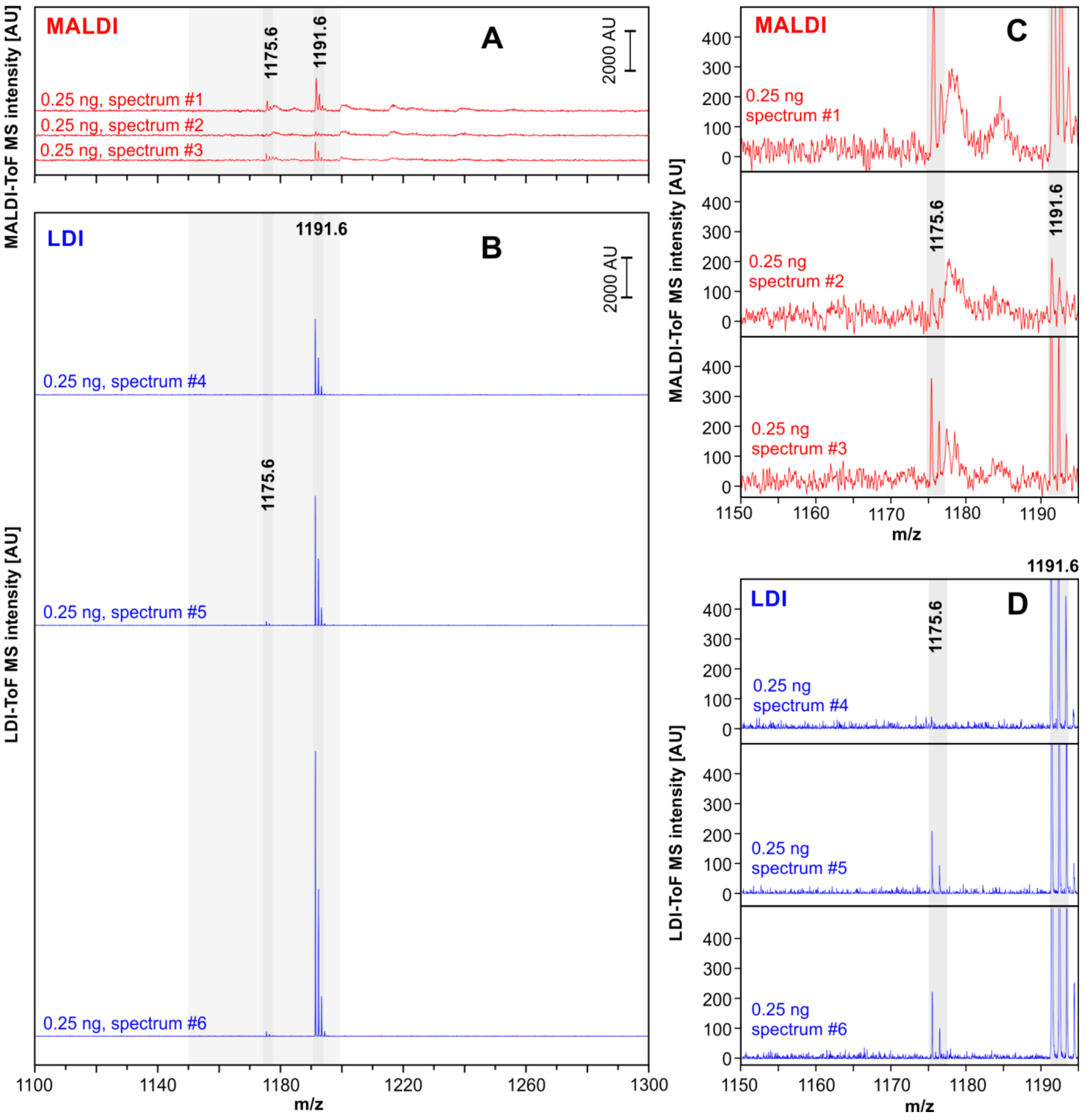

Figure SI-02. A selection of MALDI-ToF and LDI-ToF technical replicate mass spectra obtained from a commercial cereulide standard. All mass spectra were obtained in reflectron mode from a dried cereulide preparation which contained 0.25 ng of the emetic toxin per target position spot. MALDI-ToF (panels **A** and **C**, red) and LDI-ToF mass spectra (panels **B** and **D**, blue) are equally scaled to illustrate the intensity differences of the sodium $[M+Na]^+$ adduct peaks at m/z 1175.6 and the potassium $[M+K]^+$ adduct peaks of cereulide at m/z 1191.6 (see also shaded areas).

*Panels **A** and **B***: mass spectra in the m/z region of 1100 – 1300. Spectra #1-#3 (MALDI, red) and #4-#6 (LDI, blue) were selected from the series of MALDI-ToF and LDI-ToF spectra acquired to determine the LOD of cereulide (Figure 4). At 0.25 ng of the emetic toxin per target spot, both types of spectra demonstrate significant intensity variations. Cereulide adduct ion peaks in LDI spectra are generally more intense when low toxin amounts are tested.

*Panels **C** and **D**:* enlarged view of the same MALDI- or LDI-ToF mass spectra shown in the left panels. Again, spectra are equally scaled. Note the higher resolution and lower noise level of LDI-ToF mass spectra.

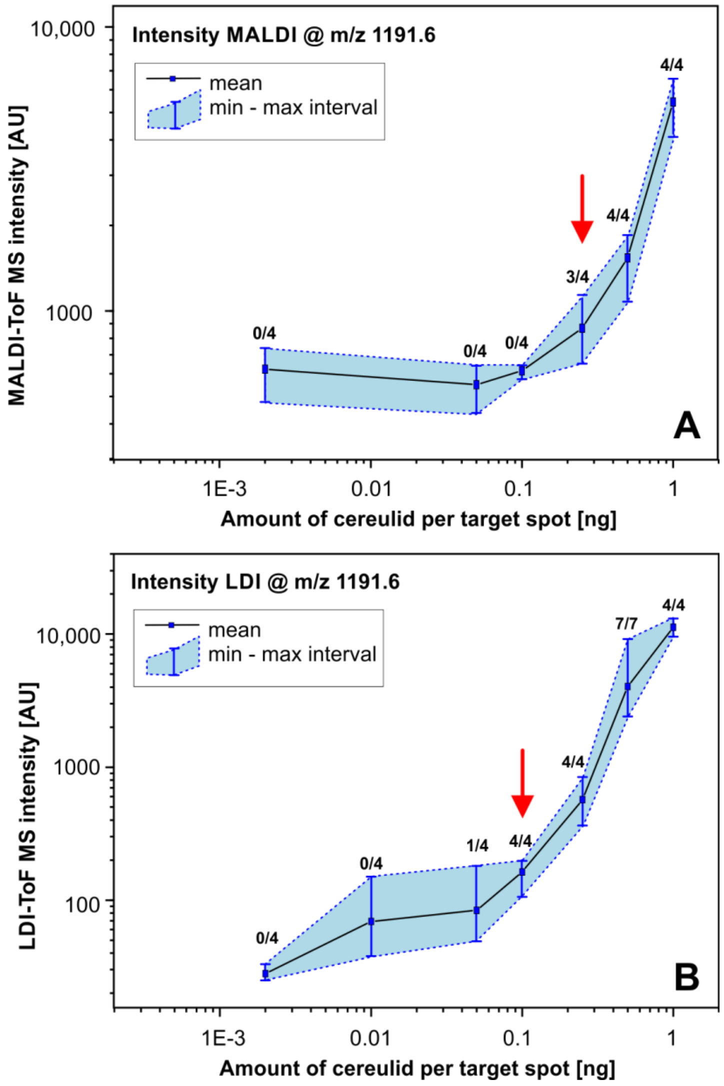

Figure SI-03. Limit of detection (LOD) of cereulide determined by MALDI- and LDI-ToF MS of ethanol wash solutions from *B. cereus* ATCC 10987 spiked by a cereulide standard. Strain *B. cereus* ATCC 10987 is an emetic-like strain without the ces gene. MALDI-ToF MS (panel **A**) and matrix-free LDI-ToF MS (panel **B**) were used for determining the LOD of cereulide from the wash solutions. Spectra were acquired under the same standardized conditions used in LOD experiments of pure cereulide (Figure 4). Data points given depict mean and min/max MS intensity values from 4 MALDI or 4 (7) LDI-ToF MS measurements at each point of the dilution series. Note the logarithmic scaling of the x- and y-axes.

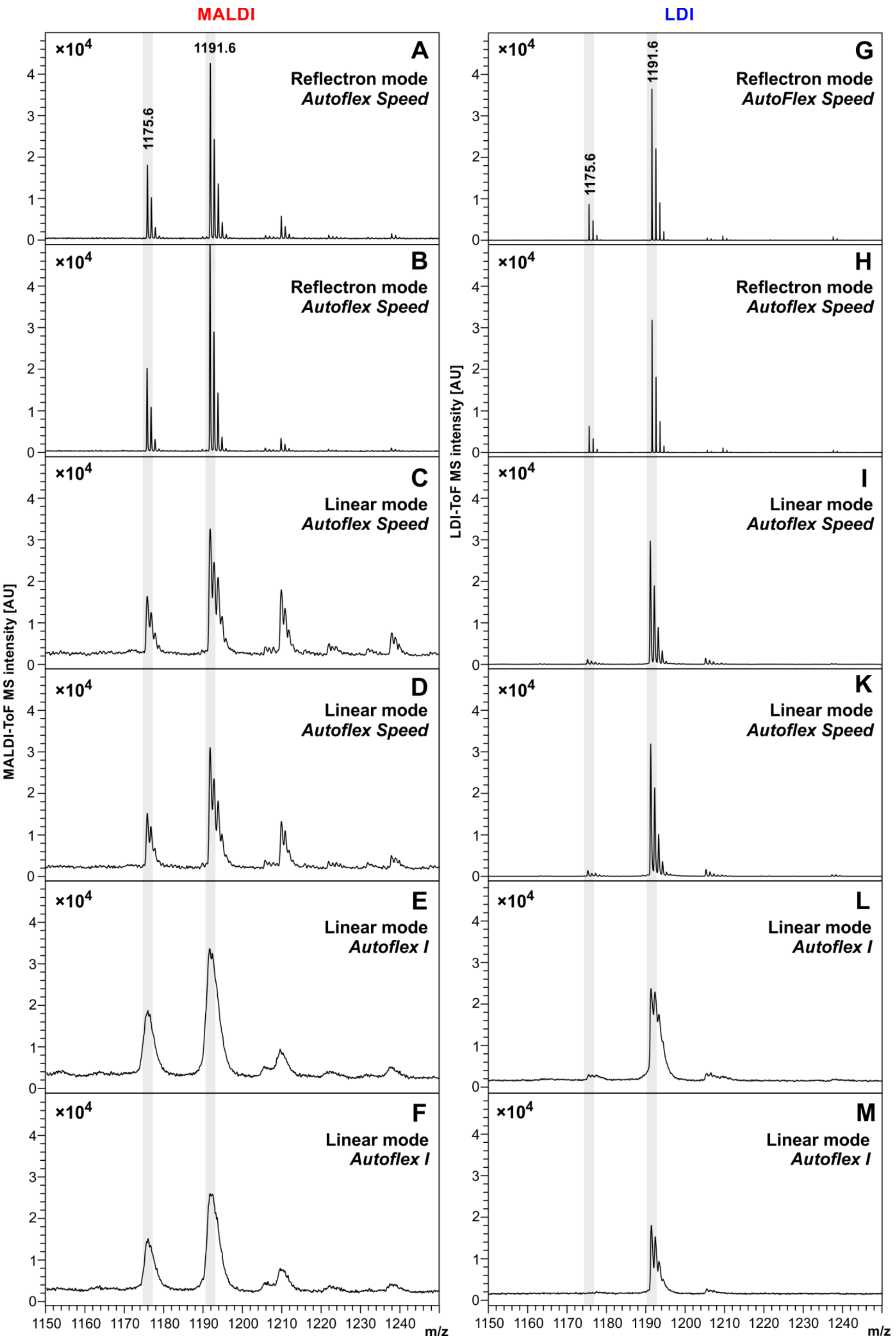
MALDI
LDI
×10^4
1191.6
1175.6
A
Reflectron mode
Autoflex Speed
B
Reflectron mode
Autoflex Speed
C
Linear mode
Autoflex Speed
D
Linear mode
Autoflex Speed
E
Linear mode
Autoflex I
F
Linear mode
Autoflex I
G
Reflectron mode
AutoFlex Speed
H
Reflectron mode
Autoflex Speed
I
Linear mode
Autoflex Speed
K
Linear mode
Autoflex Speed
L
Linear mode
Autoflex I
M
Linear mode
Autoflex I
MALDI-ToF MS intensity [AU]
LDI-ToF MS intensity [AU]
m/z
1150 1160 1170 1180 1190 1200 1210 1220 1230 1240

Figure SI-04. Direct cereulide detection by means of MALDI- (panels **A**-**F**) and LDI-ToF MS (panels **G**-**M**) in linear and reflectron measurement mode. Samples were prepared by the ethanol/FA extraction method from *B. cereus* F4810/72 cultures grown at 37°C for 24h on Caso agar. 1 µL of the raw ethanol washing solution containing 75% ethanol, non-concentrated) were directly deposited (LDI) or mixed with 1 µL HCCA matrix solution (MALDI) on target and dried subsequently on a stainless-steel target. MS measurements were carried out under highly standardized conditions in the reflectron and linear mode using an *Autoflex Speed* device equipped with sophisticated Smartbeam™ (Nd:YAG) laser technology (panels **A**-**D** and **G**-**K**), or an *Autoflex I* (N_2 laser) mass spectrometer (panels **E** & **F** and **L** & **M**), both from Bruker. Cereulide peaks are discernible at m/z 1175.6 as sodium adduct $[M+Na]^+$ and at m/z 1191.6 as potassium adduct $[M+K]^+$ of the cereulide. Peaks at m/z 1205.6 represent the potassium $[M+K]^+$ adduct of isocereulide A and/or isocereulide F.

LDI-ToF mass spectra generally exhibit higher resolution and a decreased noise level in both, linear and reflector mode measurements. Furthermore, peaks in linear mode spectra exhibit a reduced mass accuracy and a lower resolution, compared with reflector mode spectra. In addition, the limitations of entry-level equipment in terms of mass accuracy and spectral resolution are illustrated (panels **E** & **F** and **L** & **M**).
